# Motivational processes in mild cognitive impairment and Alzheimer’s disease: results from the Motivational Reserve in Alzheimer’s (MoReA) study

**DOI:** 10.1186/s12888-015-0666-8

**Published:** 2015-11-17

**Authors:** Simon Forstmeier, Andreas Maercker

**Affiliations:** Developmental Psychology, Faculty II, University of Siegen, Adolf-Reichwein-Str. 2, 57068 Siegen, Germany; Psychopathology and Clinical Interventions, Department of Psychology, University of Zurich, Binzmuehlestr. 14/17, 8050 Zurich, Switzerland

**Keywords:** Brain reserve, Mild cognitive impairment, Alzheimer’s disease, Depression, Apathy

## Abstract

**Background:**

Brain reserve, i.e., the ability of the brain to tolerate age- and disease-related changes in a way that cognitive function is still maintained, is assumed to be based on the lifelong training of various abilities. The Motivational Reserve in Alzheimer’s (MoReA) is a longitudinal study that aims to examine motivational processes as a protective factor in mild Alzheimer’s dementia (AD) and mild cognitive impairment (MCI). This paper presents the results of motivational variables, frequency of diagnoses, and prediction of global cognition as well as depression in a one-year longitudinal study.

**Methods:**

The sample consists of 64 subjects with MCI and 47 subjects with mild AD at baseline. At baseline, the physical/neurological examinations, standard clinical assessment, neuropsychological testing, and assessment of motivational variables were performed. At follow-up (FU) one year later, neuropsychological testing including cognition, functional abilities, behavioral and affective symptoms, and global clinical assessments of severity have been repeated.

**Results:**

AD cases have lower motivational capacities as measured with a midlife motivation-related occupational score and informant-reported present motivational processes, but do not differ with regard to delay of gratification (DoG) and self-reported motivational processes. DoG and delay discounting (DD) were relatively stable during the measurement interval. However, 20 % of the MCI cases converted to mild AD at FU, and 17 % of the mild AD cases converted to moderate AD. The rate of depression of Alzheimer’s disease was 9 at baseline and 21 % at FU, and the rate of apathy was 7 and 14 %, respectively. Global cognition at FU was mainly predicted by baseline global cognition but also by one of the motivational variables (scenario test). Depression at FU was predicted mainly by two motivational variables (self-reported and informant-reported motivational processes).

**Conclusions:**

This research might inform motivation-related strategies for prevention and early intervention with older people or people at risk for AD.

**Electronic supplementary material:**

The online version of this article (doi:10.1186/s12888-015-0666-8) contains supplementary material, which is available to authorized users.

## Background

One of the goals of current research on ageing and dementia is to identify the biological and psychological factors that might help individuals preserve their cognitive health into old age [1]. Comprehensive reviews identify several biological (genetic, cardiovascular, and other somatic factors) and psychological (cognitive, motivational, emotional, and social) risk factors for cognitive disorders in old age [2]. Contemporary, integrative approaches in cognitive ageing describe an interaction of cognition, motivation, and emotion [3]. There is in particular a large body of studies on motivation-cognition interactions coming from neuroscience, social and personality psychology, and aging research [4].

The present study aims to elucidate the role of motivational processes in cognitive aging and dementia. In this introduction, we first define the motivational processes under investigation; second, we describe the motivational reserve (MR) model linking motivational processes to cognitive decline and dementia; and third, we formulate the goals of the present analysis.

### Motivational processes

Motivation is an umbrella term for various processes involved in goal-directed behaviour [4]. It has been suggested by early personality psychologists [5] and more differentiated in current models of motivation [6] that two main motivational phases can be distinguished: goal setting and goal striving. Goal setting and striving are determined by rather different motivation-related constructs [7]. While goal setting is determined mainly by control and expectancy constructs [8] such as self-efficacy [9], goal striving is rather determined by volitional or self-regulatory strategies that are needed to cope with difficulties during the implementation phase such as decision regulation [10], activation regulation [11], and motivation regulation [10]. Other self-regulatory strategies are also important during goal striving, e.g., emotion and attention regulation; however, they are not motivation-related and, thus, not in focus of this study. Instead, we focus on four basic motivation-related processes relevant in goal setting and striving that are usually measured by self-report, and further variables relating to these four processes, but measured by behavioural testing, scenario tests, or an occupation-based scoring procedure.

The four basic motivation-related processes are self-efficacy (i.e., the belief in being able to master difficult demands), decision regulation (i.e., the ability to quickly come to self-congruent decisions), activation regulation (i.e., the ability to initiate a planned action), and motivation regulation (i.e., the ability to motivate oneself to persevere in the face of difficulties). These four constructs together refer to the motivational processes needed to effectively implement an intention in a self-regulated manner, and are relevant in different sub-phases in goal setting and striving [12]. Decision regulation is needed in the crossover from goal setting to goal striving; activation regulation is needed to start with an action; motivation regulation is needed to keep up with the action or to resume the action. Self-efficacy is important during the all sub-phases because it determines the amount of self-regulation effort invested as well as the perseverance.

Our measurement strategy involves different approaches in addition to self-report measures because self-reports exhibit a lower accuracy due to lack of awareness in cognitively impaired individuals [13]. All four described motivational processes are also assessed by informant-ratings as well as scenario tests. Furthermore, delay of gratification (DoG) is used which is a behavioural approach to measure motivational self-regulation [14]. Finally, occupation-based scoring procedures estimate midlife self-regulatory processes needed in a certain job [15]. Both, DoG and occupation-based scoring procedures are assumed to relate to the four basic motivation-related constructs. The methods section offers detailed descriptions of these measurement approaches.

### Motivational reserve in Alzheimer’s disease

Studies on the neuropathology of Alzheimer’s disease (AD) have repeatedly shown that many individuals with pronounced neuropathological, AD-typical changes in the brain exhibit no clinical manifestation of dementia syndrome [16]. One explanation for this finding is the concept of brain reserve [17–19]. Brain reserve can be defined as the ability of the brain to tolerate or compensate for age- and disease-related changes in a way that cognitive function is still maintained. In order to clinically manifest impairments in the cognitive and functional abilities of an individual with a larger brain reserve, it is assumed that the neuropathological damage must be more severe. The neuropathological processes seem to accumulate until they are severe enough to cross a threshold and be reflected in the clinical picture [18]. Concepts of brain reserve can explain why people differ in their reserve capacity. The extension of this threshold of clinical manifestation and the increase in brain reserve is one of the potential preventive goals for AD [20].

Theories of brain reserve assume that reserve capacity depends on a training of several abilities or the exercise of respective activities throughout life. The consequences of such a training is a more efficient use of brain networks and the compensation of affected networks [17]. It has repeatedly been demonstrated that activities stimulating the brain to a sufficient degree during the life course contribute to an increasing brain reserve. However, the question of which specific activities fulfill this criterion is not completely answered so far. Accumulating evidence has been described for the contributions of motivational, cognitive, physical, and social activities. Much research focused on cognitive activities and used the term *cognitive reserve* is [18]. Correspondingly, the authors used the term *motivational reserve* (MR) to describe the effect of motivational activities on reserve capacity [15]. MR can be defined as a set of motivational abilities or processes that provide the individual with resilience to neuropathological deterioration [15]. Motivational and cognitive reserve constitute complementary concepts in our model.

Our model presumes that activating motivational processes during the life course rises the number of synaptic connections and stimulates the development of new neurons. These neurophysiological alterations increase the efficacy of usage of relevant brain networks and enable the brain to compensate for disrupted networks (this is captured in the term ‘brain reserve’). There is a wealth of evidence that the human brain still exhibits plasticity in adult and older life [21]. The brain areas primarily involved in motivational processes are the amygdala (fear-motivated behavior), the nucleus accumbens (reward-motivated behavior), and the prefrontal cortex (regulating motivational salience and determining intensity of responding) [22, 23]. Our model includes additional factors that might mediate the effect of MR on further brain areas. These factors act by influencing stress activation, vascular risk factors, cognitive training, and emotional health [15, 24].

Several findings suggest that motivational processes predict cognitive function. A longitudinal study has shown that occupation-related motivational abilities at midlife reduce the risk of MCI by 35 % [24]. The effect on the risk of Alzheimer disease depends on the existence of an ApoE e4 allele, which was found to intensify the biochemical disturbances that are characteristic of AD including beta amyloid deposition, tangle formation, neuronal cell death, and synaptic plasticity. Midlife motivational abilities were associated with reduced risk of AD in ApoE ɛ4 carriers but not in non-carriers [24]. Two other studies support this finding using various measures of motivation-related concepts. Conscientiousness, i.e., the individual’s tendency to control impulses and be goal directed, was associated with a reduced risk of AD [25]. Purpose in life, which is associated with intentionality and goal-directed action that guides behavior, was related to a reduced risk of MCI and AD [26]. Furthermore, correlational studies have found that self-efficacy is associated with academic performance [27]. Finally, a neuroimaging study has demonstrated that internal locus of control correlates with hippocampal volume and is thus claimed to be a protective factor against age-related cognitive decline and hippocampal atrophy [28].

Cognitive reserve (CR) complements MR in our model. It has been defined as “the ability to optimize or maximize performance through differential recruitment of brain networks, which perhaps reflect the use of alternative cognitive strategies” ([29], p. 451). Education, occupation (e.g., manual vs. non-manual, psychosocial demands, and complexity of work [30], intellectual functioning (IQ and other cognitive functions) [31], and stimulating cognitive, social, and physical activities [32] are considered to index CR. Evidence for the CR hypothesis comes from cross-sectional, case–control, prospective longitudinal, and functional imaging studies [19, 33]. Prospective studies show that the risk of developing AD some time later is increased in less cognitively active adults [34–36].

The clinical implication of the motivational reserve model is that strategies for enhancing motivational abilities [37] could be adopted for use in prevention and treatment programs with older people in general or people at risk for AD (e.g., MCI). If motivational processes add to the prediction of cognitive decline, prevention programs usually including some combination of cognitive and physical training [32] should be enriched with motivational training strategies such as adaptive goal setting and self-motivation [38]. Such motivation-related interventions might also reduce neuropsychiatric symptoms of apathy and depression.

### Motivation and non-cognitive symptoms in AD and MCI

Apathy and depression are the most frequent non-cognitive symptoms in MCI and AD [39–41]. Research has shown that motivational variables can predict depression in non-demented samples. Prospective studies have found motivational self-regulation to predict depression [42] and self-efficacy to predict a variety of emotional health outcomes [9]. Correlational studies have gathered consistent support for the association of motivational variables and emotional health, e.g., mental and psychosomatic disorders [10, 37], in particular depression and anxiety [11, 43, 44].

There is evidence of an association between depressive symptoms and a higher rate of conversion form MCI to dementia [45, 46]. However, there is evidence that motivation-related symptoms of depression have a higher predictive value than the affect-related symptoms [47, 48]. Therefore, it is not surprising that apathy is also associated with a higher rate of conversion form MCI to dementia [39, 49].

Apathy can be a symptom of a neurodegenerative diseases or a syndrome in its own right [50]. As a symptom, it is frequently observed in various neuropsychiatric diseases [51]. As a syndrome, i.e., as a group of typically covarying symptoms, it has gained increasing interest in the past two decades [52]. Therefore, while current psychiatric classification systems do not provide a definition of the apathy syndrome, recently proposed diagnostic criteria define apathy as a loss or diminution of goal-directed behavior, cognition, or emotion [53]. Most current research on apathy defines it as a lack of motivation relative to the patient’s previous level of functioning or the standards of his/her age and culture [50, 54]. Although lack of motivation is still considered by many researchers to be the most important component of the apathy syndrome [52], apathy also relates to other dimensions such as interest, activity, and emotion. Therefore the current diagnostic criteria are based on the definition of apathy as lack of goal-directed behavior, cognitive activity, and emotion [53]. The goal-directed character of apathy corresponds to the goal-directed quality of motivational processes outlined above: Self-regulatory strategies (i.e., decision, activation, and motivation regulation) are needed to effectively implement a goal, in particular in the face of difficulties.

Apathy, similar as depression, can also be assumed to be predicted by motivational variables. Apathy has been linked to greater impairments in activities of daily living and a greater degree of functional decline [55] and is a distressing behavioral change for caregivers of patients with AD [56].

The distinction of apathy and depression is quite well established [57]. Although there is an overlap in symptoms (e.g., diminished interest, psychomotor retardation), there are some clear differences. Depression is characterized by dysphoric symptoms of sadness or feelings of guilt, while apathy is characterized by a lack of emotional responsiveness (emotional indifference) [58]. There are also differences in the pattern of associated cognitive deficits [59]. Additionally, neuroimaging studies have demonstrated distinct neural pathways [60].

### Goals of the present analysis

While the MoReA study includes four testing times, the goals of the present analysis refer only to baseline and one-year follow-up data and are as follows: to describe the longitudinal course of the sample including diagnoses of MCI, AD, depression and apathy and the rate of conversion from MCI to AD; to apply the motivational reserve model by comparing MCI and AD; to investigate the intercorrelations of all motivational variables (in order to know shared and unshared variance); to investigate the longitudinal course of behavioral motivation tests (delay of gratification and delay discounting); and to apply the motivational reserve model by predicting depression and global cognition at follow-up.

## Method

### Study design

The study has a prospective longitudinal design with one within-subjects variable (times of testing). The present analysis includes the baseline and one-year follow-up (FU) data. At baseline, the physical/neurological examinations, standard clinical assessment, neuropsychological testing, and assessment of motivational variables were performed. At FU, neuropsychological testing including cognition, functional abilities, behavioral and affective symptoms, and global clinical assessments of severity have been repeated. The study protocol was first approved by the Cantonal Ethics Committee of the canton Zurich, Switzerland (No. E-16/2006). This approval covers the hospitals in the canton of Zurich. The cantonal ethics committees of the cantons Aargau, Chur, St. Gallen, and Thurgau as well as the ethics committee of Caritas Socialis Vienna also approved the study protocol covering the hospitals in their respective area of responsibility, following the basic approval by the ethics committee of Zurich (see Additional file 1 for the STROBE Statement).

### Sample

The MCI subjects and mild AD patients were recruited from 14 collaborating local hospitals and clinics in the German-speaking part of Switzerland and one institution in Vienna, Austria, between 2009 and 2012. All the cooperating clinics had a department that specialized in diagnosing cognitive impairment and dementia. The subjects were referred by their general practitioners, community health services, or specialists in neurology, psychiatry, or geriatrics, and clinic-based neurological services in the greater area of Zurich, Switzerland. For inclusion in this study, the subjects had to be diagnosed as either MCI or AD at baseline (see diagnostic criteria below) and of age 55 or older. The exclusion criteria were a history of a malignant disease, severe organ failure, metabolic or hematologic disorders, neurosurgery or neurological condition such as Parkinson’s disease, epilepsy, postencephalitic and postconcussional syndrome. Written informed consent was obtained from all the participants and caregivers prior to their inclusion.

**Fig. 1:**
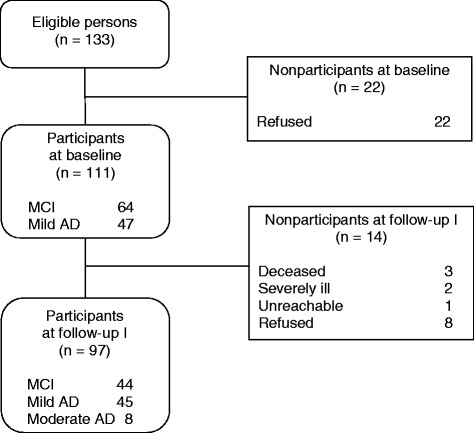
Flow chart describing sample size

Information on sample attrition is presented in Fig. 1. Overall, 133 individuals who met the inclusion and exclusion criteria were referred from the participating memory clinics to the study center. Twenty-two of these refused to participate, which resulted in 111 participants (64 with MCI and 47 with mild AD) at baseline. At follow-up 1, 14 (12.6 %) of the participants dropped out, which left 97 (87.4 %) in the study (44 with MCI, 45 with mild AD, and 8 with moderate AD). The reasons for drop-out are given in Fig. 1.

### Clinical and neuropsychological assessment

Cognitive function is primarily tested with the Consortium to Establish a Registry for Alzheimer’s Disease – Neuropsychological Assessment (CERAD-NP) [61] including the Mini Mental State Examination (MMSE) [62]. Further cognitive tests are added so there are at least two tests per domain. All the cognitive measures are given by all the sites. This battery includes:

- Episodic verbal memory: CERAD Word List Memory (learning, recall, and recognition) [63]; logical Memory subtest of the Wechsler Memory Scale-Revised (WMS-R) [64]; Digit Span Forward from the Wechsler Adult Intelligence Scale-III (WAIS-III) [65];
- Episodic non-verbal memory: Visual Reproduction subtest of the WMS-R; Figure recall score of the CERAD Constructional Praxis [66].
- Semantic memory: CERAD Animal Naming Task [67]; Modified Boston Naming Test (BNT) [68]; Controlled Oral Word Association Test [69];
- Visuoconstructive ability: Figure copy score of the CERAD Constructional Praxis [66]; Picture Completion subtest of the WAIS-III;
- Attention /cognitive speed: Trail Making Test –Part A [70]; Digit Symbol Substitution Test from the WAIS-III; Table 1 of the Stroop Color-Word Test [71];

**Table 1:** Characteristics of Participants with Mild Cognitive Impairment (MCI) and Mild Alzheimer Disease (AD) at Baseline

Characteristic	Baseline				FU	
	Total^a^ (*n* = 111)	MCI (*n* = 64)^a^	Mild AD (*n* = 47)^a^	*p* ^b^	Total^a^ (*n* = 97)	*p* ^b^
Age, years	75.3 (8.1)	73.2 (7.4)	78.0 (8.3)	.002**	76.2 (8.5)	.000***
Sex, % Female	53.2	43.8	66.0	.021*		
Education, years	12.0 (2.7)	12.3 (2.5)	11.7 (3.0)	.224		
Activities of Daily Living (Barthel)	95.7 (11.9)	99.1 (4.1)	91.0 (16.9)	.000***	95.0 (12.5)	.261
Instrumental ADL Self-report (Bayer-ADL)	2.7 (1.5)	2.4 (1.1)	3.1 (1.8)	.027*	2.7 (1.8)	.345
Instrumental ADL Informant-report (Bayer-ADL)	3.7 (2.3)	2.5 (1.5)	5.2 (2.2)	.000***	4.0 (2.4)	.002**
Cognitive status (MMSE)	25.3 (3.3)	27.1 (2.0)	22.9 (3.0)	.000***	24.9 (3.8)	.037*
Verbal Intelligence (WST)	30.6 (6.2)	31.9 (6.0)	28.7 (6.1)	.006**		
Episodic Verbal Memory^c^	−0.001 (.75)	0.42 (.61)	−0.58 (.51)	.000***	-.09 (.8)	.000***
Episodic Nonverbal Memory^c^	−0.007 (.86)	0.43 (.77)	−0.60 (.60)	.000***	.01 (.9)	.442
Semantic Memory^c^	0.005 (.82)	0.35 (.58)	−0.47 (.85)	.000***	-.23 (.9)	.000***
Visuoconstructive ability^c^	−0.01 (.89)	0.38 (.65)	−0.55 (.89)	.000***	−.04 (.9)	.409
Attention/cognitive speed^c^	0.0001 (.77)	0.28 (.66)	−0.38 (.75)	.000***	−.16 (1.1)	.033*
Executive Function^c^	−0.03 (.71)	0.28 (.66)	−0.44 (.55)	.000***	−.19 (1.1)	.065
Global Cognition^c^	−0.005 (.62)	0.36 (.45)	−0.51 (.43)	.000***	−.11 (.8)	.000***
Depression Self-report (GDS)	3.3 (2.6)	3.1 (2.7)	3.6 (2.5)	.412	2.9 (2.6)	.513
Depression Informant-report (GDS)	4.3 (3.4)	3.6 (3.3)	5.2 (3.4)	.013*	3.5 (2.9)	.283
Depression Clinical Rating (CSDD)	3.0 (3.9)	2.9 (3.4)	3.1 (4.5)	.904	3.8 (3.9)	.112
Apathy Self-report (AES)	31.5 (6.6)	30.8 (7.2)	32.5 (5.6)	.186	31.0 (6.9)	.273
Apathy Informant-report (AES)	37.2 (11.3)	35.7 (11.7)	39.3 (10.5)	.101	38.0 (11.7)	.138
Apathy Clinical Rating (AES)	35.0 (7.5)	33.7 (7.9)	36.9 (6.6)	.027*	34.6 (8.8)	.292
Neuropsychiatric Inventory Total	6.8 (10.7)	6.3 (9.7)	7.5 (11.9)	.561	6.9 (9.5)	.910
Activities (self-report)	32.3 (10.3)	34.1 (9.7)	29.7 (10.7)	.027*	32.9 (11.5)	.980
Perceived Social Support	4.2 (.54)	4.1 (0.6)	4.2 (0.5)	.445	4.2 (.6)	.617

*Abbreviations*: *MMSE* Mini-Mental State Examination, *WST* Wortschatztest (Vocabulary Test), *GDS* Geriatric Depression Scale, *CSDD* Cornell Scale for Depression in Dementia, *AES* Apathy Evaluation Scale

^a^Unless otherwise specified, the data represent the mean (SD)

^b^P value of t or *χ* ^2^ tests. *p < .05, **p < .01, ***p < .001

^c^Average of *z* scores. See Methods section for included tests
- Executive function: Task switching: Trail Making Test –Part B [70]; Inhibition of prepotent responses: Stroop Color-Word Test [71]; Updating working memory: Digit Span Backward from the WAIS-III.

Because of the wide range of cognitive function, individual cognitive tests are subject to floor and ceiling artifacts [72]. To minimize such artifacts and other sources of measurement error, we calculated composite measures for the cognitive domains and a global cognition composite score. By converting the component tests to z scores by using the baseline mean and SD of all study participants and averaging the z scores, the composite measures were constructed.

Non-cognitive symptoms are assessed using the following measures:

- Activities of daily living (ADL) were assessed by the Barthel Index [73], which is the standard ADL measure in German speaking countries. Instrumental activities of daily living (IADL) was assessed by the Bayer-ADL [74], which is a 25-item self- and informant-rated, internationally used questionnaire with established validity and reliability.
- Depression and apathy were assessed by the short form of the Geriatric Depression Scale (self- and informant-reported) (GDS) [75], the Cornell Scale for Depression in Dementia (clinician-rated) (CSDD) [76], and the Apathy Evaluation Scale (self-, informant- and clinician-rated) (AES) [77]. The diagnosis of depression was based on the provisional diagnostic criteria for depression of AD [78]. The diagnosis of apathy was based on the criteria proposed by Starkstein et al. [79].

Finally, global clinical assessments of severity were performed using the Clinical Dementia Rating (CDR) scale [80].

### Diagnostic procedures

The *diagnoses of probable AD* were made according to the DSM-IV-TR criteria for AD and the National Institute of Neurological and Communicative Disorders and Stroke/Alzheimer’s Disease and Related Disorders Association (NINCDS-ADRDA) criteria for AD [81]. The NINCDS-ADRDA criteria require a history of cognitive decline and evidence of impairment in memory and at least one other cognitive domain. *Possible AD* cases (in NINCDS-ADRDA terminology) were also included, i.e., persons who met these criteria and also had another condition thought to be contributing to cognitive impairment. Only AD cases with a mild dementia severity were included, and this was determined by the Clinical Dementia Rating (CDR) scale (i.e., scores of 1) [80] and the Mini Mental State Examination (MMSE) (i.e., scores of 18–28) [62].

*MCI* was defined according to the international consensus criteria [82]: (a) absence of dementia as diagnosed by the DSM–IV criteria (MMSE ≥ 24); (b) cognitive decline, i.e., according to self and/or informant report and impairment on objective tasks and/or evidence of decline over time on objective cognitive tasks; (c) preserved basic activities of daily living and not exceeding minimal impairment in complex instrumental functions (CDR ≤ 0.5); (d) at least mild impairment in one of the following cognitive domains: memory, language, praxis, executive function, and attention.

Consensus conferences across the participating sites were held to assure that the diagnostic criteria were applied similarly across sites.

### Assessment of motivational variables

Our measurement strategy involved four different approaches.

1. Midlife motivation-related occupational score. The basis of this retrospective estimation of MR is the main occupation of the subject and a sample of Occupational Information Network (O*NET) variables [15]. The O*NET is the official occupational classification system of the US Department of labor [83, 84]. Beside a hierarchically structured lexicon of occupations, the O*NET includes a large database of work and worker characteristics associated with each occupation. This database is the result of a continuing, large-scale research program over recent decades. In the O*NET data collection program, samples of workers in each occupation were assessed using questionnaires including items on work and worker characteristics. For the goal orientation variable, for example, workers were asked “How important is organizing, planning, and prioritizing work (i.e., developing specific goals and plans to prioritize, organize, and accomplish your work) to the performance of your current job?” Answers were coded on a 5-point scale.

The procedure of estimating former motivational abilities consists of three steps. First, the subjects and their informants were asked to name the occupations they held (a) in their first job after finishing their education that was held for at least 1 year, (b) in their (maximum of four) longest held jobs, and (c) in the last job of their professional life. For each job, information was collected on the start and finish dates, job title, and major activities and duties. Second, O*NET occupational codes corresponding to the main occupation were assigned to each participant. Information on the subjects’ major occupational activities and duties was crucial for their coding to O*NET occupations. The coders compared the activities and duties the participants reported with those provided in the O*NET classification system. The occupation that exhibited the best match was selected. Therefore, this coding procedure is also largely applicable to Swiss occupations. Each participant’s occupational information was coded independently by two coders. When a disagreement between the coders was found, the participant’s answers and the O*NET job descriptions were reexamined and the coding alternatives was discussed until a consensus was reached. The subjects who had been housewives for the longest period of their life were classified according to their second-longest held job. Third, the value of two O*NET variables that comprises motivational processes (“goal orientation” and “action planning”) were assigned to the participants. These two variables have been selected in a previous study, see [15] for more information. A composite measure was calculated after converting the two O*NET variables to z scores by using the baseline mean and SD of all the study participants and averaging the z scores.

2) Behavioral tasks: Delay of Gratification and Delay discounting. Delay of gratification (DoG) tasks have been used extensively to measure motivational self-regulation in children [85]. We developed a DoG for Adults (DoG-A) task [86] that used four rewards: snacks (8 items), hypothetical money (8 items), real money (1 item), and magazines (1 item). A total score was calculated from all items. The framing story was that the participant and interviewer draw a pawn on a board through the streets of a city. On each field, a card was drawn that asks the participant for a decision. The participant was informed that his preferences and interests are being assessed. In the previous session, various sweets and snacks of small size had been offered, and the participant was asked to choose his two favorites. The same was done to find their favorite magazine.

These are simple decisions that individuals with MCI and mild AD are capable of making. Ten percent of the participants in our pilot study exhibited a mild cognitive impairment but had no difficulties with the DoG-A [86]. Support for its validity comes from correlations with delay discounting and self-reported motivation regulation as well as zero correlations with cognitive tests of verbal intelligence, memory, processing speed, verbal fluency, and executive function [86].

The delay discounting rate was assessed using the Delay Discounting questionnaire [87]. The participants were presented with a fixed set of 27 choices between smaller, immediate money rewards and larger, delayed rewards. For example, the participants were asked “Would you prefer CHF 68 today or CHF 69 in 92 days?” The 27 items were grouped into three magnitude categories: small (32–44 Swiss Francs, CHF), medium (CHF 63–76), and large (CHF 95–107). The discounting rates were estimated on the basis of the pattern of 27 choices. Discounting curves have been shown to be best described by a hyperbolic decay function. The discounting rate *k* increases with the individual’s preference for immediate rewards. Therefore, a higher discounting rate *k* can be interpreted as lower self-control or higher impulsiveness. The validity of *k* as a behavioral measure of self-control/impulsiveness is indicated by its correlation with impulsiveness [88, 89].

3) Motivational Scenario Test (MST). The MST is a newly developed measure of motivational processes [Forstmeier & Maercker: The Motivational Reserve Scenario Test (MRST), in preparation]. The scenario technique has previously been used in research on self-regulation [90]. Four short scenarios were described to the participant in either oral or written form. In each of the scenarios, the exertion of motivational abilities was required (e.g., motivation, decision, activation regulation, and self-efficacy). The subjects were instructed to immerse themselves in this scenario and to imagine being in this situation. To support the identification with the scenario, the subject was asked to state his or her thoughts and emotions that appeared (these were not subjected to analysis). Then, they were asked what they would do in this situation. Four items with a 5-scale answer format (1 = I would definitely not do this, 3 = neutral, 5 = I would definitely do this) had to be answered; two of these reflected high motivational ability and two reflected low motivational ability (inversely rated). Scores from 4 to 20 were possible in each scenario. Our pilot study has shown that participants with mild cognitive impairments were able to state their preferred reaction in these imagined situations. Participants with mild AD were observed in the present study to be also capable to imagine the situations and answer the simple questions.
4) Self-report and informant-report questionnaires. Four variables (motivation regulation, decision regulation, activation regulation, and self-efficacy) have been assessed from two perspectives (self-report and informant-report) and two time points (present and retrospective), which resulted in four versions each. Informant-report questionnaires that estimate the participant’s premorbid motivational abilities during the age of 30–50 are modifications of established self-report measures. We chose a cut off of 30 because individuals still experience considerable personality change in their 20’s. The items of the informant-report versions were slightly rephrased compared to the original questionnaires to assess the same aspect retrospectively and from the informant’s point of view (e.g., “I energetically pursue my goals” - “He/she energetically pursued his/her goals.”). Four scales were used:

Two scales of the Volitional Components Questionnaire (VCQ) [10] were used to assess motivation regulation (e.g., “He/she could usually motivate his/herself quite well if his/her determination to persevere weakened;” Cronbach’s alpha = .87) and decision regulation (e.g., “When he/she thought about doing or not doing something, he/she usually arrived at a decision quickly;” alpha = .69).

We used the locomotion scale of the Locomotion and Assessment Questionnaire (LAQ) [11] to measure activation regulation. The scale consists of 10 statements regarding activating oneself or starting an action (e.g., “When he/she decided to do something, he/she couldn’t wait to get started;” alpha = .74).

The General Self-Efficacy scale (GSE) [91] assesses the “broad and stable sense of personal competence to deal effectively with a variety of stressful situations” ([91], p. 243). Participants rated 10 items (e.g., “He/she was confident that he/she could deal efficiently with unexpected events”) on a 4-point scale (alpha = .91).

Whenever possible, both the informant retrospective version and the self-report retrospective version were used (e.g., “I energetically pursued my goals;” alphas between .66 and .84). The subjects also completed the original version, i.e., referring to their current motivational abilities (e.g., “I energetically pursue my goals;” alphas between .66 and .89). Finally, the informant completed a version that refers to the present (e.g., “He/she energetically pursues his/her goals;” alphas between .75 and .91).

### Assessment of cognitive reserve

CR is measured using an O*NET-based estimate of premorbid cognitive abilities and the three typical indices of education, verbal intelligence, and stimulating activities [18].

1. Midlife cognition-related occupational score. We described the O*NET in the previous section. We selected four variables that correlated significantly with verbal intelligence (measured with a vocabulary test) but not with established self-report measures of motivational abilities: selective attention, recognizing problems, assessing performance, and social perceptiveness [15]. The value of these O*NET variables was assigned to the participants. A composite measure was calculated after the four O*NET variables were converted to z scores by using the baseline mean and SD of all the study participants and averaging the z scores.
2. Education. Education was measured in terms of the highest year of schooling completed. Persons with a university (master’s) degree were coded as having completed 18 years of education, and persons with a PhD or MD were coded as having completed 21 years of education regardless of their actual years in school.
3. Verbal intelligence. Premorbid IQ was estimated by using a verbal intelligence test. Verbal intelligence, as measured with a German vocabulary test (Wortschatztest, WST) [92], remains relatively preserved in old age and in the early stages of AD, and thus, constitutes a good estimate for premorbid IQ [93]. The test consists of 42 lines with six words each. One of the words in a line is real and five are nonsense. In each line, the participants were asked to identify the real word. Difficulty increased from line to line.
4. Stimulating activities. A questionnaire that was filled in by the patient and the informant assessed activities, in a way similar to [36, 94]. The patients and informants were asked to rate how often the subjects participated in 21 common activities during the past twelve months on a 6-point scale (1 = every day or about every day; 2 = several times a week; 3 = once a week; 4 = several times a month; 5 = several times a year; 6 = never). The eight physical activities included walking, bicycling, swimming, muscle training, game sports, gymnastics, dancing, and housekeeping or gardening. The five cognitive activities included reading books/newspapers, writing, studying, working crossword puzzles, and playing cards or board games. The four creative activities included playing a music instrument, painting, cooking, and sewing/knitting/crocheting. Finally, the four social activities included meeting with people, attending the theatre/concerts/exhibitions, political engagement, and social engagement (e.g., church). Five mean scores were be calculated: four category scores and one total score (alpha = .65 and .66 for the self-report and informant-report versions, respectively).

### Procedure

Individuals who fulfilled the selection criteria for the AD or MCI group as well as their caregivers/informants were informed about the study by the therapeutic staff at the clinical institutions or by the researcher involved in the study, and given a leaflet that explained the objectives and procedures. The patients and informants who expressed interest in participation provided written consent to meeting with a project psychologist or receiving a phone call from him.

The participants’ current health history was reviewed with the patients and/or the informant at FU, and this included neuropsychological testing and an assessment of behavioral measures of motivational processes. Examiners were blinded to previously collected data. In the MCI group, the diagnosis of dementia was reappraised. The first meeting had a duration of approximately 60 min, and the second meeting had a duration of approximately 90 min. If the meeting took longer than this or the patient/informant arrived at his attention limit, a further meeting was arranged. Parallel to the assessment of the patient, the caregiver was interviewed and completed questionnaires.

### Statistical analysis

A principal components analysis (with varimax rotation) was performed in order to reduce the number of motivational variables in the planned analyses. Seven factors have been extracted by visual inspection of the scree plot, with the following variables loading mainly on these factors (see Additional file 2: Table S1): 1) motivation-related occupational score; 2) DoG and DD; 3) motivational scenario test; 4) the four self-report measures (presence); 5) the four self-report measures (retrospective); 6) the four informant-report measures (presence) as well as three informant-report retrospective measures (motivation regulation, decision regulation, and self-efficacy); and 7) activation regulation in its informant-report (retrospective) version. The seven factors account for 72.3 % of the total variance. Since DD, with money as reward, is a subcomponent of the DoG measure, we decided to use only DoG in the following analyses. Composite scores for self-report presence, informant-report presence, and self-report retrospective were calculated by converting the component tests to z scores and averaging the z scores. The three informant report retrospective variables were not included in the composite score in order to have a pure presence-related score. The retrospective informant-report score includes only the activation regulation variable.

The group (AD and MCI) differences in the demographic and clinical measures were tested by using *χ*^2^ and t-tests. Pearson product–moment (and Spearman rank) correlations were used to test the associations among the motivational variable measures and their relation to neuropsychological performance in the AD and MCI groups. To adjust for 21 correlation tests (Tab. 4), the critical alpha-level was reduced to .0024. Two separate multiple hierarchical regression analyses were conducted to examine the baseline predictors of global cognition and depression at FU.

## Results

### Sample characteristics

Characteristics of the sample and the descriptive data at baseline are given in Table 1. The 111 participants (64 with MCI and 47 with mild AD at baseline) had a mean age of 75 (age range 55–94) and a mean duration of education of 12 years. Fifty-three percent of the participants were women. The participants with AD were significantly older and more functionally and cognitively impaired than those with MCI. They also tended to have more neuropsychiatric symptoms, but this is only significant for informant-rated depression as well as clinician-rated apathy.

### Diagnoses of MCI, AD, depression, and apathy

Table 2 presents the diagnoses of MCI and AD at baseline and follow-up. From the 64 MCI cases at baseline, 13 (20.3 %) converted to mild AD at follow-up. From the 47 mild AD cases at baseline, 32 (68.1 %) remained mild AD and 8 (17.0 %) progressed to moderate AD.

**Table 2:** Diagnoses of Mild Cognitive Impairment (MCI) and Mild Alzheimer Disease (AD) at Baseline and Follow-up

Baseline	Follow up			
	MCI (*n* = 44)	Mild AD (*n* = 45)	Moderate AD (*n* = 8)	Drop-out (*n* = 14)
MCI (*n* = 64)	44 (68.8 %)	13 (20.3 %)	0 (0 %)	7 (10.9 %)
Mild AD (*n* = 47)	0 (0 %)	32 (68.1 %)	8 (17.0 %)	7 (14.9 %)

*Note*: *χ* ^2^ = 58.8, *p* < .001

Structured clinical interviews were used to establish the rate of depression of Alzheimer’s Disease (DAD) and apathy at baseline and follow-up. Nine percent of the total baseline sample received the diagnosis of DAD; there was no difference between the MCI and AD cases (9.4 % vs. 8.5 %, *χ*^2^ = .02, *p* = 1.00). Then, 20.7 % were diagnosed with DAD at follow-up, and again there was no difference between the MCI and AD cases (20.3 % vs. 21.3 %, *χ*^2^ = .02, *p* = 0.90). There was a significant difference between the test times (McNemar: *p* = .007).

Apathy was diagnosed in 7.2 % of the total sample at baseline, without a difference between the MCI and AD cases (7.8 % vs. 6.4 %, *χ*^2^ = .08, *p* = 0.77). Slightly more, i.e., 14.4 % of the total sample received an apathy diagnosis at follow-up, with significantly more apathy in the AD group (7.8 % vs. 23.4 %, *χ*^2^ = 5.34, *p* = 0.02). However, the difference between test times was not significant (McNemar: *p* = 0.13).

### Motivational variables: intercorrelations and comparison of MCI and AD

In terms of the O*NET major occupational groups, the largest group (21 %) had worked in office and administrative support occupations, 14 % had worked in management occupations, 11 % had worked in sales and related occupations, 10 % had worked in production occupations, and less than 5 % had worked in each of the other occupational groups (see Table 3).

**Table 3:** Frequency of Midlife Occupational Categories (O*NET-SOC Major Groups) and Mean Motivation- and Cognition-Related Occupational Abilities

Characteristic	Total	MCI	AD	Mot. abilit.	Cog. abilit.
Management Occupations (11)^a^	14.4 %	18.8 %	8.5 %	0.66 (.54)	0.65 (.24)
Business and Financial Operations Occupations (13)	2.7 %	4.7 %	0 %	0.85 (.58)	0.12 (.44)
Computer and Mathematical Occupations (15)	0 %	0 %	0 %	-	-
Architecture and Engineering Occupations (17)	3.6 %	6.3 %	0 %	1.20 (.59)	0.64 (.72)
Life, Physical, and Social Science Occupations (19)	0 %	0 %	0 %	-	-
Community and Social Services Occupations (21)	2.7 %	1.6 %	4.3 %	0.27 (.74)	1.07 (.51)
Legal Occupations (23)	0 %	0 %	0 %	-	-
Education, Training, and Library Occupations (25)	2.7 %	0 %	6.4 %	0.79 (.09)	0.63 (.47)
Arts, Design, Entertainment, Sports, and Media Occupations (27)	3.6 %	3.1 %	4.3 %	−0.15 (.37)	0.38 (.31)
Healthcare Practitioners and Technical Occupations (29)	2.7 %	3.1 %	2.1 %	0.87 (.35)	1.48 (.52)
Healthcare Support Occupations (31)	3.6 %	6.3 %	0 %	−0.64 (.83)	0.10 (.70)
Protective Service Occupations (33)	1.8 %	1.6 %	2.1 %	0.63 (.79)	1.27 (.01)
Food Preparation and Serving Related Occupations (35)	4.5 %	3.1 %	6.4 %	−1.22 (.96)	−0.74 (.60)
Building and Grounds Cleaning and Maintenance Occupations (37)	1.8 %	1.6 %	2.1 %	−0.75 (.98)	−1.14 (.50)
Personal Care and Service Occupations (39)	3.6 %	3.1 %	4.3 %	−1.23 (.78)	0.49 (.07)
Sales and Related Occupations (41)	10.8 %	12.5 %	8.5 %	0.05 (.58)	−0.03 (.26)
Office and Administrative Support Occupations (43)	20.7 %	17.2 %	25.5 %	−0.42 (.49)	−0.64 (.29)
Farming, Fishing, and Forestry Occupations (45)	2.7 %	0 %	6.4 %	−0.93 (.80)	−0.71 (.39)
Construction and Extraction Occupations (47)	1.8 %	1.6 %	2.1 %	0.75 (1.11)	−0.26 (.48)
Installation, Maintenance, and Repair Occupations (49)	1.8 %	3.1 %	0 %	0.83 (.34)	0.26 (1.11)
Production Occupations (51)	9.9 %	10.9 %	8.5 %	−0.08 (.81)	−0.61 (.84)
Transportation and Material Moving Occupations (53)	1.8 %	1.6 %	2.1 %	0.10 (1.12)	0.49 (1.55)
Military Specific Occupations (55)	0 %	0 %	0 %	-	-
*χ* ^2^ or F tests and p value		*χ* ^2^ = 29.1, *p* = 0.10		F = 6.2, *p* < 0.001	F = 10.4, *p* < 0.001

*Abbreviations*: *MCI* Mild Cognitive Impairment, *AD* Alzheimer’s disease

^a^The numbers in brackets represent the number of the O*NET-SOC major groups

The distribution of occupational groups did not differ for the MCI and AD groups (*χ*^2^ = 29.1, *p* = 0.10), but some of the occupations that show relatively high mean motivation-related occupational abilities are more frequent in the MCI participants (e.g., management, business/financial, architecture/engineering, and installation occupations). The 23 occupational categories differ in their mean motivation-related (F = 6.2, *p* < 0.001) and cognition-related (F = 10.4, *p* < 0.001) occupational abilities. There are many occupations that require either more motivation-related abilities (e.g., business/financial, architecture/engineering¸ protective service, construction, and installation occupations) or more cognition-related abilities (e.g., community/social, arts/entertainment, healthcare, and personal care occupations).

Table 4 presents the mean, standard deviations, and comparisons between the MCI and AD participants for the motivational variables at baseline. Generally, the MCI and AD participants had similar motivational capacities. However, the AD cases had lower motivational capacities as measured with the motivation-related occupational score and the informant-reported (present) motivational processes composite.

**Table 4:** Motivational Variables: Intercorrelations and Comparison of Mild Cognitive Impairment (MCI) and Mild Alzheimer Disease (AD) (*n* = 111)

Characteristic	(1)	(2)	(3)	(4)	(5)	(6)	(7)
(1) Midlife motivation-related occupational score		-.09	.00	.09	.03	.22*	−.05
(2) Delay of Gratification			.08	.05	.11	.11	.00
(3) Motivation Scenario Test				.32**	.18	.19*	.08
(4) Self-reported motivational processes (presence)					.24*	.60**	.16
(5) Informant-reported motivational processes (presence)						.20*	.35**
(6) Self-reported motivational processes (retrospective)							.12
(7) Informant-reported motivational processes (retrospective)							
Total sample: M (SD)	−.002 (.88)	2.10 (1.35)	49.72 (5.77)	.00 (.77)	.00 (.84)	.00 (.73)	52.10 (8.17)
MCI (*n* = 64): M (SD)	.15 (.90)	2.20 (1.37)	50.16 (5.32)	.04 (.69)	.19 (.80)	−.01 (.75)	51.99 (8.08)
AD (*n* = 47): M (SD)	−.22 (.80)	1.96 (1.33)	49.09 (6.38)	−.06 (.87)	−.26 (.82)	.01 (.70)	52.25 (8.38)
p (of t)	.034*	.35	.35	.50	.005**	.87	.87

*p < .05, ** < .0024. To adjust for 21 correlation tests, the critical alpha-level is reduced to .0024

As expected, there were several meaningful and significant intercorrelations between the motivational variables, although many of them were small to medium. The motivation-related occupational score correlated with retrospectively self-reported motivational processes (r = .22, *p* < .05), which lost its significance when adjusting for multiple tests. The Motivation Scenario Test correlated with self-reported (present) motivational processes (r = .32, *p* < .0024; adjusted for multiple tests). Self-reported motivational processes in its present and retrospective versions correlated highly significant (r = .60, *p* < .0024) as well as informant-reported motivational processes in its present and retrospective versions (r = .35, *p* < .0024). Finally, DoG correlated with delay discounting (r = −.23, *p* < .05) and self-reported (present) motivation regulation (r = .22, *p* < .05), however, without significance when adjusting for multiple tests (not shown in Tab. 4). The patterns of correlations are very similar for the subsamples of MCI and AD cases (see Additional file 3: Tables S2 and S3).

### Longitudinal analysis of delay of gratification (DoG) and delay discounting (DD)

DoG was relatively stable during the one-year measurement interval (means in the total sample: 2.1 and 1.9 at baseline and follow-up, respectively; main effect Time: F = .89, *p* = .39; analyses controlled for age, sex, and gender). DoG tended to be lower in the AD than the MCI cases, but this difference was not statistically significant (main effect Diagnostic category: F = 3.38, *p* = .07; Time x Diagnostic category interaction: F = .59, *p* = .45).

DD was also relatively stable (means in the total sample: .037 and .054 at baseline and follow-up, respectively; main effect Time: F = .65, *p* = .42); however, there was a significant interaction effect for DD (F = 6.60, *p* = .01): the AD cases had a reduced self-control (higher DD) after one year (.037 vs. .082), which was not the case for the MCI cases (.037 vs. .036). The main effect of the diagnostic category was not significant (F = .11, *p* = .74).

### Prediction of global cognition and depression at follow-up

Several bivariate correlations and two multiple hierarchical regression analyses were conducted to examine the baseline predictors of global cognition (average of z-transformed scores of tests in six cognitive domains) and depression at follow-up (Tab. 5). The following baseline variables correlated significantly with global cognition at FU: age (*r* = −.50, *p* < .001); global cognition (*r* = .88, *p* < .001); activities (*r* = .33, *p* = .001); Motivation Scenario Test (*r* = .21, *p* = .02); and informant-reported motivational processes (*r* = .34, *p* = .001). In a regression analysis, global cognition at FU was predicted by education (*β* = .12, *p* = .04), baseline global cognition (*β* = .91, *p* < .001), depression (*β* = .12, *p* = .05), and the Motivation Scenario Test (*β* = .11, *p* = .05).

**Table 5:** Summary of Bivariate Correlations and Separate Multiple Hierarchical Regression Analyses Predicting Depression and Global Cognition at Follow-up (*N* = 97)

	Global cognition^a^ at FU			Depression at FU		
	*r*	*β*	*p*	*r*	*β*	*p*
Epidemiological variables						
Age	−.50***	.06	.34	.18*	.15	.18
Sex (1 = male, 2 = female)	−.04	−.03	.67	−.08	.05	.68
Education (years)	.03	−.12	.04*	.08	.08	.46
Cognition-related occupational score	.15	−.02	.74	−.01	.03	.84
Clinical/cognitive variables at baseline						
Global cognition	.88***	.91	<.001***	−.14	.05	.70
Depression	.13	.12	.05*	.35***	.15	.20
Activities (self-report)	.33***	.04	.53	−.12	−.01	.91
Perceived Social support	.06	−.02	.67	−.24**	−.16	.11
Instrumental ADLs	−.09	.06	.37	.35***	.23	.07
Motivational variables at baseline						
Motivation-related occupational score	.09	−.04	.55	.06	.08	.56
Delay of Gratification	.14	−.03	.52	−.16*	−.05	.59
Motivation Scenario Test	.21*	.11	.05*	−.12	.19	.08
Self-reported motivational processes	−.03	−.06	.33	−.34***	−.24	.03*
Informant-reported motivational processes	.34***	.05	.38	−.43***	−.25	.02*
Corrected R^2^		.78			.26	
F and p		24.53	<.001		3.36	<.001

*Note:* *FU* Follow-up after one year

*p < .05

**p < .01

***p < .001

^a^Global cognition: composite measures for all tests in all cognitive domains (through converting the component tests to z scores by using the baseline mean and SD of all study participants and averaging the z scores

The following baseline variables correlated significantly with depression (CSDD) at FU: age (*r* = .18, *p* = .04); depression (*r* = .35, *p* < .001); perceived social support (*r* = −.24, *p* = .01); instrumental ADLs (*r* = .35, *p* < .001); delay of gratification (*r* = −.16, *p* = .05); self-reported motivational abilities (*r* = −.34, *p* < .001); and informant-reported motivational processes (*r* = −.43, *p* < .001). In a regression analysis, depression at FU was predicted by only two motivational variables, i.e., self-reported motivational processes (*β* = −.24, *p* = .03) and informant-reported motivational processes (*β* = −.25, *p* = .02).

## Discussion

The current study’s main focus lies on the prediction of disorder- and age-related cognitive impairment with motivational processes by applying our model of motivational reserve capacity [15] in a longitudinal study.

### Prevalence of MCI, AD, depression, and apathy

Previous epidemiological studies found annual conversion rates from MCI to AD to be between 2.3 and 19.1 % [95]. Thus, the conversion rate of 20.3 % lies at the upper border of this interval. This somewhat higher conversion rate compared to other studies might be foremost attributed to the fact that a certain time period passed between the diagnosis of MCI and the inclusion in the study, which resulted in more than one year between the diagnosis of MCI and the follow-up.

Structured clinical interviews were used to establish the rate of depression of Alzheimer’s disease and apathy at baseline and follow-up. The diagnosis of depression was based on the provisional diagnostic criteria for depression of AD [78]. We found a prevalence of 9 at baseline and almost 21 % at follow-up; the patients with MCI and AD did not differ in their depression rate. The rate at follow-up is similar to that found previously in a sample of patients with probable AD (27.4 %) [96]. In other studies, higher rates of depression in AD were found, namely between 44 and 53.5 % [97–99]. The higher rates in these studies might be attributed to the fact that these samples had been intentionally selected to increase the frequency of depression, as noted by [97]. The depression rate at baseline is relatively low (9 %) and increased to 21 % during one year. This indicates that depression changes as the disease progresses. Previous research has shown that depression typically appears in mild to moderate stages and becomes less prevalent in more severe stages [100]. Thus, we would expect an inverted U-curve when looking at the development from MCI to severe dementia.

The rate of apathy measured by a structured clinical interview [79] was 7.8 and 7.8 % in the MCI patients and 6.4 and 23.4 % in mild AD patients at baseline and follow-up, respectively. This is in accordance with the previously found 19 % in a sample of patients with probable AD [79]. Higher prevalence rates can be found when rating instruments and cut-off scores are used [101]. This can also be demonstrated in the present study: While the rate of apathy as diagnosed with a structured clinical interview was 7.2 % in the total sample, it was 9.9 %, 43.1, and 30.9 % using the apathy evaluation scale (self, informant, and clinical report, respectively) using recommended cut-off scores [77]. In addition, the prevalence increases with the severity of dementia [102]. This increase in prevalence can particularly be observed in patients diagnosed with mild AD at baseline in this study, but apathy is relatively stable in the MCI patients.

### Testing of motivational reserve predictions

Individuals with MCI and AD do not differ with regards to delay of gratification, scenario test, and self-report measures. However, there are two interesting differences, which support our MR model predictions. The AD cases have lower values than the MCI cases in the midlife motivation-related occupational score. This means that the MCI cases had occupations that required more motivation-related occupational abilities (e.g., management, business/financial, architecture/engineering, and installation occupations) more often than did the AD cases. The same advantage of MCI cases over AD cases with regard to the occupation-based motivational variable was also observed in two other samples in Germany [24] and the United States [103].

In addition, the AD cases have a lower informant-reported (present) motivation processes score than the MCI cases. In contrast, the AD cases rated themselves to be not less self-regulated than the MCI cases. In other words, only the caregivers of the AD patients and not the patients themselves rate the patient’s motivational abilities to be lower than the caregivers of the MCI patients. The sources of this difference might be twofold [104]. First, the motivational abilities of the AD cases might be truly lower than those of the MCI cases, and the caregivers observe this decline but the patients themselves do not observe it because of a reduced disease awareness (“anosognosia”), which results in similar self-report values [105]. Second, the AD cases might be more burdened and depressed by the larger impairment of AD vs. MCI cases and see the abilities of the patient more negatively than they are (“caregiver rating bias”) [106]. Both processes can act together. A consequence of these considerations is that not only should self-reports of the patient’s symptoms and abilities be interpreted with caution but also informant-reports.

Behavioral tests such as the delay of gratification and the delay discounting test gain a greater importance in this sample because of the reduced validity of the self- and informant-reports. In our sample, the people with MCI and mild AD do not differ with regard to these variables. The question arises how long DoG and DD remain stable when the disease progresses. We discuss this topic below.

As expected, there are a multitude of meaningful and significant intercorrelations between the MR variables (in the whole sample and both subsamples) that are in line with previous publications that use non-impaired samples. Midlife motivation-related occupational abilities correlate with retrospectively self-reported motivational processes. This was also found in a sample of cognitively healthy individuals [15]. Delay of gratification correlated with delay discounting and self-reported (present) motivation regulation. This correlation pattern was also found in a sample of cognitively healthy individuals [86], which resulted in the interpretation that DoG represents a behavioral test of self-motivation.

### Behavioral motivational tests in longitudinal course

DoG and DD represent behavioral tests of self-control. High self-control is reflected in high DoG and low DD. Compared with identical test procedures for cognitively healthy older people who exhibit an average DoG score of 2.4 and an average DD score of 0.027 [86], self-control in persons with MCI or mild AD seems to be slightly lower.

The present data give a first answer to the question of how stable DoG and DD are during one year in the course of disease-related cognitive impairment. Both DoG and DD are relatively stable during the one-year measurement interval as the non-significant main effect Time reveals. However, there is a significant interaction effect for DD (but not DoG): the AD cases have a reduced self-control (i.e., higher DD) after one year, but this is not the case for the MCI cases.

The fact that DoG is relatively stable during one year but cognitive functions declined significantly requires an explanation. This result is not surprising if DoG is considered a motivational function that is different from cognitive functions [108, 109]. However, it is surprising if it is considered a rather cognitive, i.e., cold executive function [109]. It has long been shown that executive functions decline as early as the mild stages of AD [110] and decline during the course of AD [112]. If DoG is a cold executive function, i.e., cognitive at its core, we would expect it to decline over a year’s time. In contrast, motivational-affective functioning declines more slowly during the course of Alzheimer’s dementia and is rather preserved in its early stage [112, 113]. The present results suggest that DoG includes motivational rather than cognitive processes. This is also supported by the correlation with self-reported motivation processes, which has also been shown in a sample of cognitively healthy older people [86].

In contrast, delay discounting was higher (i.e., self-control was lower) after one year in the sample of patients with AD. Delay discounting includes hypothetical money rewards that require more cognitive resources (e.g., working memory and mathematical operations) than the real rewards in DoG. Therefore, one can argue that delay discounting captures cold executive functions, while DoG covers hot executive functions.

### Motivation predicting subsequent cognitive status

The research question was whether motivational variables are negatively associated with cognitive decline and impairment. Our results provide further evidence that this is the case. To our knowledge, this is the first study to show motivational variables as predictors in the course of cognitive impairment. In addition to age, baseline cognition, and activities, two motivational variables (the Motivation Scenario Test and informant-reported motivational processes) correlated with global cognition at follow-up, of which only the scenario test remained a significant predictor in the regression analysis. In contrast to our previous findings, the occupation-based measure of midlife motivational abilities was not associated with global cognition at follow-up. In a sample of cognitively healthy individuals, the same measure was associated with cognitive function [15]. In addition, this measure was associated with a reduced risk of MCI and a reduced risk of AD in ApoE ɛ4 carriers in a longitudinal study [24]. Future analyses of this longitudinal studywill reveal whether the midlife motivation-related occupational score will predict cognitive decline over the course of several years rather than cognitive status one year after baseline.

Two other studies support the evidence that motivational variables are associated with cognitive decline. Conscientiousness, which is a related construct that can be defined as the tendency to control impulses and to be goal directed, has been found to be associated with a lower risk of MCI and AD in a prospective study [25]. A high level of conscientiousness was associated with an 89 % reduction in the risk of AD. In addition, high purpose in life, which results in intentionality and motivated action, was also associated with a reduced incidence of MCI and AD in a prospective study [26]. Although conscientiousness and purpose in life are not core motivational variables, they may translate into an increased sense of intentionality or goal-directed striving and, thus, support the findings of the present study that better motivational abilities at baseline are associated with better cognitive function at follow-up.

### Motivation predicting subsequent depression

We expected the motivational variables to be negatively associated with depression, when controlling for other potential predictors. Our results extend the previously found association between motivational variables and emotional health in a sample of cognitively impaired individuals. In fact, two motivational variables remain the only predictors of depression at follow-up in a regression analysis. Age, baseline depression, perceived social support, and instrumental ADLs showed significant bivariate correlations but lost their significance in the regression analysis.

Most research on the association between motivational processes and emotional health was done with cognitive healthy samples. For instance, prospective studies demonstrated that motivational self-regulation predicts depression [42] and self-efficacy predicts emotional health [9]. Correlational studies support the association of motivational processes and emotional health, e.g., psychiatric and psychosomatic disorders [10, 37, 114], in particular anxiety and depression [11, 27].

Although there is support for the association between motivational variables and future depression, motivational variables have rarely been included in epidemiologic studies on predictors of depression in the general older population or in cognitively impaired older people. The established risk factors of depression in non-demented older people are bereavement, sleep disturbance, disability, prior depression, and female gender, as demonstrated by a meta-analysis [115]. In a sample of non-demented individuals, of whom 25 % exhibited MCI, only functional impairment, MCI, and smoking were significant predictors in a fully adjusted analysis [116]. Some of these predictors were also found in our study. Functional impairment was associated with depression in the univariate analysis but missed significance in the multivariate analysis. Baseline depression was associated in only the univariate analysis. Because baseline depression and motivational variables were strongly associated with each other, only motivational variables survived the regression analysis. Future epidemiologic studies should include motivational variables as predictors of depression in the general population and in demented populations.

### Strengths and limitations

The current study tries to extend previous research on brain reserve or cognitive reserve in predicting age- and dementia-related cognitive decline by introducing a broad range of motivational variables. Cognitive and motivational status was assessed by behavioral or performance measures and informant ratings as well as standard self-report ratings. The study predictions were based on a theoretical model [15] that has gained some support in methodologically diverse studies [24, 86, 103].

The results should be interpreted in light of several limitations. The analyses were performed with only two of the four planned assessment times of this longitudinal study. Data of the third and fourth assessment time are not available yet. Thus, only a brief period during the course of a cognitive impairment is covered. In particular, the longitudinal analysis of delay of gratification and delay discounting as well as the prediction of cognition and depression will gain importance when a longer time period is covered. It can assumed that delay of gratification, which is relatively stable after one year, will eventually decrease with cognitive decline but will do so slower.

The sample is rather small compared to many other large-scale longitudinal studies. However, this is the first study that focuses on motivational variables as predictors of cognitive decline and emotional health in the cognitively impaired, and we hope this will pioneer other, larger studies on this topic.

The correlations between the motivational variables vary between small-to-medium and large effects. Large correlations (i.e., around .5) were found only between the instruments that used the same procedure (e.g., self-report) and the same source (e.g., regarding self and present). The low-to-medium correlations may be attributable to behavioral and self-report measures that capture *different facets* of a related construct, namely motivational self-regulation. This phenomenon is also found in other areas of psychological measurement; for example, implicit and explicit measures of motives are relatively independent [117]. Furthermore, self-report measures are more prone to cognitive decline and a social desirability response bias. Because behavioral and self-report measures of motivation tap different aspects of the same construct to a certain extent, it is important to use both approaches in research to ensure the comprehensive assessment of the construct.

We used the current criteria of depression in AD [78] and apathy [53] with adequate structured clinical interviews [79]. However, these DSM-like criteria do not have a long tradition in Alzheimer’s and aging research and, thus, have yet to gain a large dissemination. More research will reveal the best symptom criteria for depression in AD and apathy and how changing criteria will influence study results such as those of the present study.

## Conclusions

This research brings the episodic knowledge that the personal abilities colloquially called *will power, self-discipline*, or *self-regulation* protect against cognitive decay on a scientific basis. The MoReA study aims to investigate motivational factors that contribute to the development of AD, including its cognitive decline as well as depressive symptoms and apathy. Motivational factors were neglected in previous research. Once relevant motivational factors have been identified and shown to be important in the cognitive decline of AD, new and promising strategies for prevention and early intervention can be developed and applied. Our own previous research has shown that there are effective strategies for enhancing motivational competencies that have been applied to various psychiatric disorders such as depression and anxiety [37]. These strategies can be adopted for use in prevention and psychotherapy programs at midlife, with older people in general, or with people at risk for AD (e.g., with MCI). Enhancing motivational abilities might also increase the effectiveness of pharmacological and psychosocial interventions. By targeting the neuropsychiatric symptoms of apathy and depression, caregiver burden might be reduced. The results of previous studies on brain reserve have been used to make recommendations about lifestyle and activities in late life to decrease the risk of AD [32]. The possible results of our study would add recommendations about changes in motivational self-regulation in later life [38].

## Additional files

Additional file 1:
**STROBE Statement—Checklist of items that should be included in reports of cohort studies.** (PDF 28 kb) Additional file 2: Table S1. Factor Analysis of Motivation-Related Constructs. (PDF 52 kb) Additional file 3: Tables S2 and S3. Motivational Variables: Intercorrelations in the subsample of individuals with Mild Cognitive Impairment (MCI) and in the subsample of individuals with Mild Alzheimer Disease (AD). (PDF 68 kb)

## Abbreviations

AD: Alzheimer’s disease
ADL: Activities of daily living
AES: Apathy Evaluation Scale
BNT: Boston Naming Test
CDR: Clinical Dementia Rating
CERAD-NP: Consortium to Establish a Registry for Alzheimer’s Disease – Neuropsychological Assessment
CR: Cognitive reserve
CSDD: Cornell Scale for Depression in Dementia
DAD: Depression of Alzheimer’s Disease
DD: Delay discounting
DoG: Delay of gratification
DoG-A: Delay of gratification test for adults
DSM-IV-TR: Diagnostic and statistical manual of mental disorders iv – text revision
FU: Follow-up
GDS: Geriatric depression scale
GSE: General Self-Efficacy scale
IADL: Instrumental activities of daily living
IQ: Intelligence quotient
LAQ: Locomotion and Assessment Questionnaire
MCI: Mild cognitive impairment
MMSE: Mini Mental State Examination
MoReA: Motivational reserve in Alzheimer’s study
MR: Motivational reserve
MST: Motivational Scenario Test
NINCDS-ADRDA: National Institute of Neurological and Communicative Disorders and Stroke/Alzheimer’s Disease and Related Disorders Association
O*NET: Occupational Information Network
VCQ: Volitional Components Questionnaire
WAIS-III: Wechsler Adult Intelligence Scale-III
WMS-R: Wechsler Memory Scale-Revised
WST: Wortschatztest.
