# Supplementary material for: Motivational processes in mild cognitive impairment and Alzheimer’s disease: results from the Motivational Reserve in Alzheimer’s (MoReA) study
Source: BMC Psychiatry. 2015 Nov 17;15:293. doi: 10.1186/s12888-015-0666-8 (PMC4650956; doi:10.1186/s12888-015-0666-8)
Supplement: Additional file 2: Table S1. — Factor Analysis of Motivation-Related Constructs. (PDF 52 kb) [file 12888_2015_666_MOESM2_ESM.pdf]

Table S1. Factor Analysis of Motivation-Related Constructs (N = 97)

Variable	Occup. score	Behav. tasks	Scen. test	Self-rep. pres.	Self-rep. retr.	Inform.-report	Activ. reg inform. retro
Midlife motivation-related occupational score	**.799**						
Delay of Gratification		**.790**					
Delay Discounting		**-.753**					
Motivation Scenario Test			**.640**				
Self-reported Motivation Regulation (presence)				**.825**			
Self-reported Decision Regulation (presence)			.313	**.689**	.365		
Self-reported Activation Regulation (presence)			.354	**.697**			.425
Self-reported Self-efficacy (presence)				**.789**			
Self-reported Motivation Regulation (retrospective)	.329			.463	**.538**		
Self-reported Decision Regulation (retrospective)					**.853**		
Self-reported Activation Regulation (retrospective)			.472		**.552**		.459
Self-reported Self-efficacy (retrospective)				.409	**.781**		
Informant-reported Motivation Regulation (presence)						**.825**	
Informant -reported Decision Regulation (presence)						**.820**	
Informant -reported Activation Regulation (presence)			.310			**.728**	
Informant -reported Self-efficacy (presence)						**.890**	
Informant -reported Motivation Regulation (retrospective)			-.306	.493		**.525**	.326
Informant -reported Decision Regulation (retrospective)	-.310				.412	**.533**	
Informant -reported Self-efficacy (retrospective)	-.360				.328	**.502**	
Informant -reported Activation Regulation (retrospective)							**.852**
Eigenvalue	1.192	1.338	1.964	2.312	2.527	3.667	1.451
% of Total Variance	5.96	6.69	9.82	11.56	12.63	18.33	7.26
Total Variance	72.25%						
