# Supplementary material for: Motivational processes in mild cognitive impairment and Alzheimer’s disease: results from the Motivational Reserve in Alzheimer’s (MoReA) study
Source: BMC Psychiatry. 2015 Nov 17;15:293. doi: 10.1186/s12888-015-0666-8 (PMC4650956; doi:10.1186/s12888-015-0666-8)
Supplement: Additional file 3: Tables S2 and S3. — Motivational Variables: Intercorrelations in the subsample of individuals with Mild Cognitive Impairment (MCI) and in the subsample of individuals with Mild Alzheimer Disease (AD). (PDF 68 kb) [file 12888_2015_666_MOESM3_ESM.pdf]

Table S2. Motivational Variables: Intercorrelations in the subsample of individuals with Mild Cognitive Impairment (MCI) (n = 64).

Characteristic	(1)	(2)	(3)	(4)	(5)	(6)	(7)
(1) Midlife motivation-related occupational score		-.12	.01	.06	-16	.17	-.06
(2) Delay of Gratification			.12	.01	-03	.02	.06
(3) Motivation Scenario Test				.29*	.04	.25*	.02
(4) Self-reported motivational processes (presence)					.22	.52**	.08
(5) Informant-reported motivational processes (presence)						.21	.41**
(6) Self-reported motivational processes (retrospective)							.15
(7) Informant-reported motivational processes (retrospective)							

* *p* < .05, ** < .0024. To adjust for 21 correlation tests, the critical alpha-level is reduced to .0024.

Table S3. Motivational Variables: Intercorrelations in the subsample of individuals with Mild Alzheimer Disease (AD) (n = 47).

Characteristic	(1)	(2)	(3)	(4)	(5)	(6)	(7)
(1) Midlife motivation-related occupational score		-.11	-.04	.12	.16	.36*	-.04
(2) Delay of Gratification			.01	.09	.24	.25	-.07
(3) Motivation Scenario Test				.36*	.32*	.14	.15
(4) Self-reported motivational processes (presence)					.24	.72**	.25
(5) Informant-reported motivational processes (presence)						.22	.32*
(6) Self-reported motivational processes (retrospective)							.07
(7) Informant-reported motivational processes (retrospective)							

* *p* < .05, ** < .0024. To adjust for 21 correlation tests, the critical alpha-level is reduced to .0024.
